# Testosterone replacement therapy in insulin‐sensitive hypogonadal men restores phosphatidylcholine levels by regulation of arachidonic acid metabolism

**DOI:** 10.1111/jcmm.15392

**Published:** 2020-06-03

**Authors:** Giuseppina Fanelli, Antonio Belardo, Rocco Savino, Sara Rinalducci, Lello Zolla

**Affiliations:** ^1^ Department of Ecological and Biological Sciences (DEB) University of Tuscia Viterbo Italy; ^2^ Department of Medical and Surgical Sciences Magna Graecia University Catanzaro Italy; ^3^ Department of Science and Technology for Agriculture, Forestry, Nature and Energy (DAFNE) University of Tuscia Viterbo Italy

**Keywords:** arachidonic acid metabolism, hypogonadism, lipidomics, testosterone replacement therapy

## Abstract

Male hypogonadism is notoriously associated with altered lipid metabolism. In this study, we performed an untargeted mass spectrometry–based profiling of plasma lipids from twenty healthy and twenty hypogonadal men before and after testosterone replacement therapy (TRT) for 60 days. Results demonstrated that hypogonadism was associated with a significant increase in sphingomyelin (SM), whereas phosphatidylcholine (PC) was mainly cleaved by activated phospholipase‐A2 into lysophosphatidylcholine (LPC). In hypogonadal patients, arachidonic acid (AA), also produced through the latter cleavage, was prevalently bio‐transformed into leukotriene B4 (LTB4) and not into endoperoxides from which prostaglandins and thromboxanes are derived. Interestingly, upon testosterone treatment SM, PC and LPC returned to levels similar to controls. Also, AA was newly converted into prostaglandin‐A2, thromboxane‐A2 and 5(S)‐hydroxyeicosatetraenoic acid (HETE), suggesting that testosterone probably plays a role in controlling hypogonadal alterations above reported.

## 1 INTRODUCTION

Low testosterone levels are one of the main traits of hypogonadism, causing a multidimensional metabolic syndrome characterized by obesity, diabetes, hypertension and dyslipidaemia.^1^ Hence, a better understanding of molecular mechanisms that contribute to the regulation of lipid metabolism is crucial to establish new therapeutic strategies for hypogonadism. In recent years, lipidomics has emerged as a powerful tool in the field of biomedical research for the comprehensive characterization of lipid species and the investigation of the complex metabolic networks of lipids in a biological system.^2^ On the basis of homeostasis model assessment index (HOMAi), hypogonadal men can be categorized into hyper‐insulinaemic (also defined as insulin‐resistant) patients and normo‐insulinaemic (also defined as insulin‐sensitive) patients. Clearly, in these two sub‐groups, the inflammatory mediators increase differently and interfere with insulin signalling in different ways. This is probably not only reflected at the carbohydrate metabolic level as already discussed in two previous works,^3^, ^4^ but also lipid‐related metabolisms are expected to be altered due to the demonstrated relationship between insulin resistance and fat gain.^5^ Thus, by an untargeted LC‐MS/MS platform coupled with LipidSearch™ software analysis, here we profile, for the first time, plasma lipids in hypogonadal insulin‐sensitive (IS) men. This strategy aims to provide novel molecular endophenotypes potentially useful to refine phenotypic information in hypogonadal patients and healthy individuals.

## 2 MATERIALS AND METHODS

### 2.1 Study participants and therapy

Twenty healthy and twenty hypogonadal male subjects were enrolled in our study. Hypogonadism was diagnosed using clinical symptoms, including erectile dysfunction, decreased libido, and/or decreased energy as well as evidence of low serum testosterone (≤8 nmol/L). Hypogonadism‐affected patients were included only if they had HOMAi < 2.5. The hypogonadal patients were treated with a 2% testosterone gel preparation for 60 days. The gel was formulated to have a similar application and appearance. All patients gave their informed consent before participating in the study. The research was approved by the local Institutional Ethical Committee Board.

### 2.2 Metabolite and lipid measurements

Human blood samples were collected after overnight fasting and processed according to ethical guidelines and standards of practice. EDTA‐plasma was prepared by 10‐minute centrifugation at 4°C and 3000 *g*. Ultra‐performance liquid chromatography coupled to electrospray ionization mass spectrometry (UHPLC‐ESI‐MS) was used to explore the plasma lipid and metabolite profiles. Methods of lipid and metabolite extraction, along with instrument setting for their analysis, are described in detail in supplementary material.

## 3 RESULTS

**FIGURE 1:**
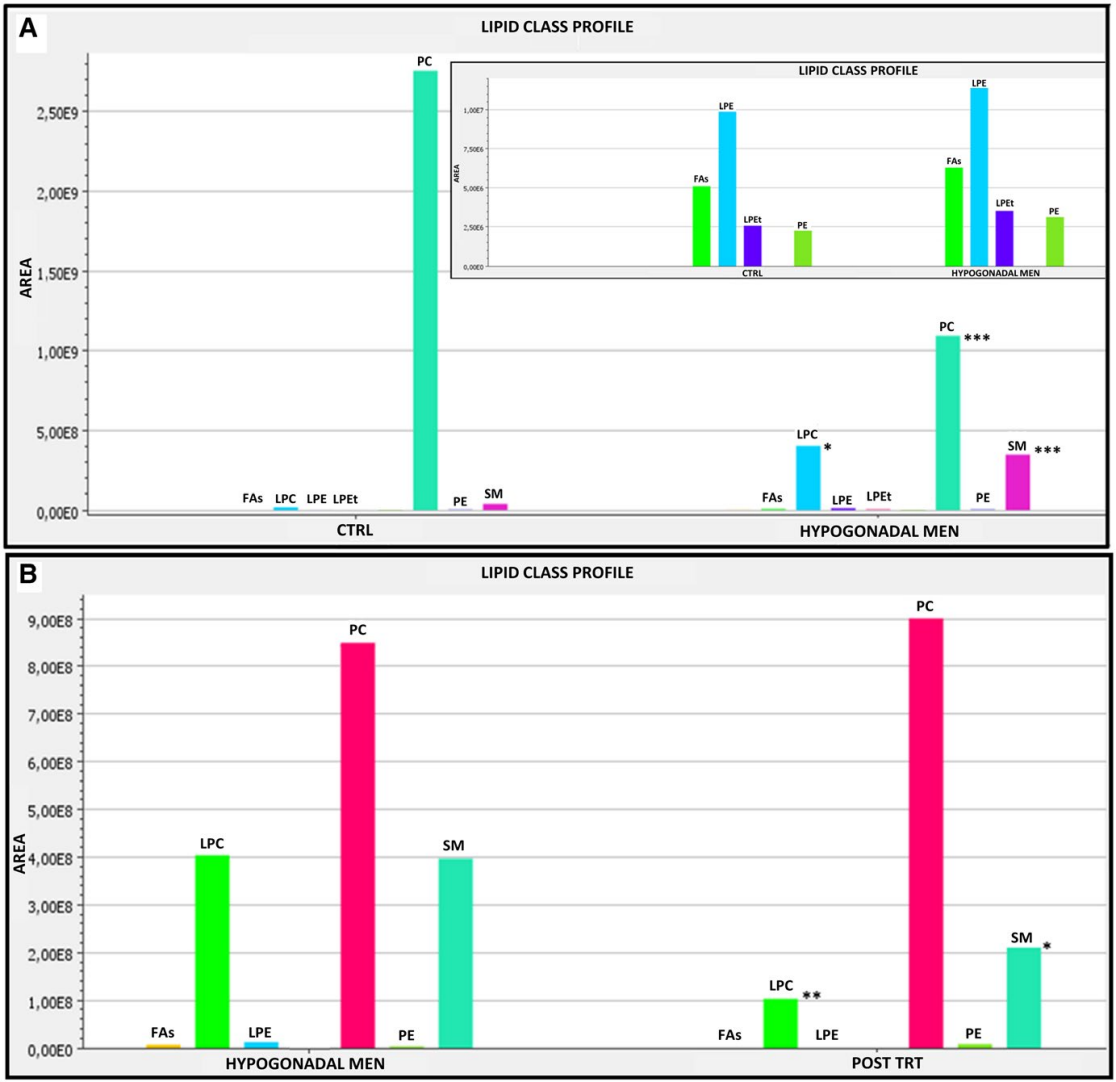
Analysis of plasma lipid classes. Panel A, lipid class profiles as a result of control and hypogonadal men comparison. Inset shows a zoom of lipid classes with low intensities. Panel B, lipid class profiles from hypogonadal men before and after testosterone replacement therapy (TRT). FAs, fatty acids; LPC, lysophosphatidylcholine; LPE, lysophosphatidylethanolamine; LPEt, lysophosphatidylethanolamine‐t; PC, phosphatidylcholine; PE, phosphatidylethanolamine; SM, sphingomyelin. Asterisks indicate statistical significance (Student's *t* test: *P < 0.05, **P < 0.01, ***P < 0.001)

**FIGURE 2:**
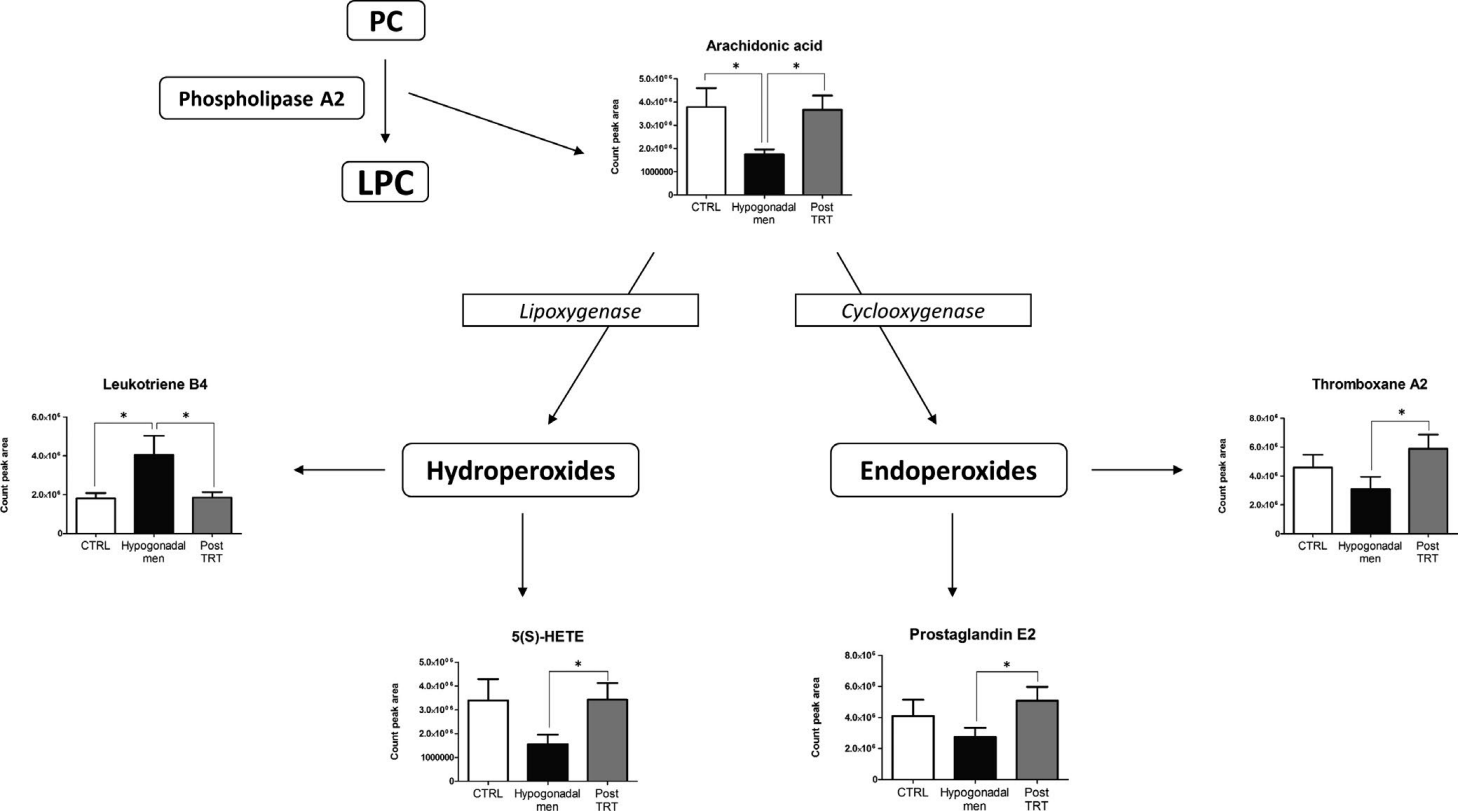
Analysis of key metabolites from the arachidonic acid pathway. Data are expressed as mean ± SD (n = 20). Asterisks indicate statistically significant differences between groups (Student's *t* test: *P < 0.05). 5(S)‐HETE, 5(S)‐hydroxyeicosatetraenoic acid; LPC, lysophosphatidylcholine; PC, phosphatidylcholine; TRT, testosterone replacement therapy

Baseline characteristics and endocrine variables of control and IS hypogonadal patients are shown in Table S1. LC‐MS/MS lipidomics of plasma from these subjects showed interesting lipid differences. The reliability and reproducibility of the whole analysis were evaluated by the quality control (QC) samples used during the experiment. Quality control samples were closely clustered in the PCA scores plot (Figure S1); thus, no QC‐based drift correction or data cleaning was performed. A total of 77 lipid species (CV < 20%) were identified in both hypogonadism‐affected and control men. These observed lipids could be sub‐divided into seven classes: fatty acids (FAs), PC, LPC, lysophosphatidylethanolamine (LPE), lysophosphatidylethanolamine‐t (LPEt), phosphatidylethanolamine (PE) and SM. As shown in Figure 1A, levels of FAs, LPE and PE were similar between hypogonadal and control subjects, whereas hypogonadism condition was associated with increased SM and a higher bio‐transformation of PC into LPC. Switching from PC to LPC also releases arachidonic acid (AA), that in healthy men is metabolized through cyclooxygenase‐ and lipoxygenase‐mediated pathways into bioactive eicosanoid lipids: prostaglandins, thromboxanes, HETE and leukotrienes (Figure 2). In contrast, in hypogonadal patients, AA was preferentially bio‐transformed into LTB4, more so than in 5(S)‐HETE. Moreover, decreased concentrations of thromboxane‐A2 and prostaglandin‐E2 were found, suggesting a weakening of the cyclooxygenase‐dependent pathway in hypogonadism (Figure 2).

After 60 days of TRT, no statistically significant differences were revealed in BMI, triglycerides, LDL and HDL (Table S1), whereas the levels of SM and PC, as measured by LC‐MS/MS, returned to similar values of the control group (Figure 1B). The same was registered for plasma concentration of AA, 5(S)‐HETE, thromboxane‐A2 and prostaglandin‐E2; also, the levels of LTB4 were re‐established after testosterone administration (Figure 2).

## 4 DISCUSSION

Our investigation revealed that plasma levels of PC were lower in hypogonadal patients, whereas a significant increase of LPC was observed, this being related to higher activity or overexpression of phospholipase‐A2 (PLA2) as reported by Keleşoğlu et al^6^ Greater concentrations of LPC increase cardiovascular risk via its effects on lipid metabolism^6^; conversely, PC contributes to increase FA oxidation or metabolism and lowers the cholesterol absorption in the gastrointestinal tract.^7^ The PLA2‐mediated cleavage of PC into LPC also releases AA, which in IS hypogonadal men was preferentially bio‐transformed through the lipoxygenase pathway into LTB4 rather than 5(S)‐HETE, in spite of thromboxane‐A2 (TXA2) and prostaglandin‐E2 (PGE2) production, that significantly decreased. Higher levels of leukotrienes were previously related to testosterone deficiency and to the onset of disorders such as asthma, a common long‐term inflammatory disease often associated with hypogonadism.^8^, ^9^ Reduction of PGE2, a powerful vasodilator, may instead represent one of the causes of the hypogonadism‐associated erectile dysfunction in men. In fact, impairments of the cyclooxygenase‐dependent AA metabolism were associated with pathogenesis of both endothelial dysfunction and augmented vasoconstriction in penile arteries.^10^ In our research, hypogonadal patients showed augmented concentrations of SM that is converted into ceramide and PC. As it is well known that ceramide regulates testosterone production in Leydig cells,^11^ high levels of SM may inhibit testosterone biosynthesis. Higher plasma SM levels were also found in human familial hyperlipidaemias, especially in familial hypercholesterolaemia, suggesting that such a condition can be a risk factor for atherosclerosis,^12^ commonly observed in hypogonadal men.^1^

Interestingly, 60 days of testosterone replacement therapy (TRT) were not sufficient to improve triglycerides, HDL or LDL plasma levels in hypogonadal patients, whereas PC and LPC returned to values similar to controls, indicating that testosterone can directly or indirectly control bio‐transformation of PC into LPC. In agreement with a recent report by DeBoer et al^13^ TRT also reduced LTB4 production from AA, so that a relationship between low testosterone and accumulation of the LTB4 metabolite exists. The plasma values of 5(S)‐HETE were also re‐established post‐TRT, suggesting a feedback correlation between testosterone and 5(S)‐HETE, probably related to the evidence that 5(S)‐HETE improves testosterone secretion by Leydig cells.^14^ In response to testosterone administration, thromboxane‐A2 (TXA2) and prostaglandin‐E2 (PGE2) were produced again. Accordingly, model studies on rats showed that testosterone therapy increases aortic TXA2 receptor density and responsiveness.^15^ Finally, a restoration of SM levels post‐TRT was recorded, confirming previous studies showing that supplementation of testosterone for hypogonadism improved cardiovascular health.^16^

In summary, the present study showed for the first time that changes in the level of specific lipid species (especially PC, LPC and SM) and in the two cascade pathways of AA metabolism can be the cause of some clinical consequences of hypogonadism. These alterations were significantly restored upon testosterone administration after only 60 days. On the contrary, this time was not sufficient to re‐establish other lipid classes, such as HDL, LDL, cholesterol and triglycerides.

## CONFLICT OF INTEREST

All authors declare that there is no duality of interest associated with their contribution to this manuscript.

## AUTHOR CONTRIBUTIONS

LZ and RS made study concept and design; GF involved in data acquisition and analysis; GF, AB, SR and RS involved in data interpretation; SR and LZ drafted the manuscript; and all authors critically revised the manuscript.

## Supporting information

Supplementary Material

## Data Availability

The data that support the findings of this study are available on request from the corresponding author.
