# Supplementary material for: Testosterone replacement therapy in insulin‐sensitive hypogonadal men restores phosphatidylcholine levels by regulation of arachidonic acid metabolism
Source: J Cell Mol Med. 2020 Jun 3;24(14):8266–9. doi: 10.1111/jcmm.15392 (PMC7348168; doi:10.1111/jcmm.15392)

**SUPPLEMENTARY MATERIAL**

**Testosterone replacement therapy in insulin-sensitive hypogonadal men restores phosphatidylcholine levels by regulation of arachidonic acid metabolism**

Giuseppina Fanelli^1^, Antonio Belardo^1^, Rocco Savino^2^, Sara Rinalducci,^1,*^ and Lello Zolla ^3,*^

*^1^Department of Ecological and Biological Sciences (DEB), University of Tuscia, Viterbo, Italy. ^2^Department of Medical and Surgical Sciences, Magna Graecia University, Catanzaro, Italy. ^3^Department of Science and Technology for Agriculture, Forestry, Nature and Energy (DAFNE), University of Tuscia, Viterbo, Italy.*

***Corresponding authors:** Lello Zolla ([zolla@unitus.it](mailto:zolla@unitus.it)); Sara Rinalducci ([sara.r@unitus.it](mailto:sara.r@unitus.it))

**Materials and Methods**

**Chemicals.** LC/MS grade acetonitrile, isopropanol, chloroform, methanol, ammonium acetate, formic acid and water (≥98% chemical purity) were purchased from Sigma Aldrich.

**Lipid extraction.** Lipids were extracted with chloroform:methanol (2:1, v/v) according to the original Folch procedure. Briefly, ice-cold methanol and chloroform were added directly to biological fluid. The suspension was vortexed at room temperature for 20 min. After the addition of water, used to separate the aqueous and organic layer, the suspension was incubated on ice for additional 10 min. Samples were centrifuged and then the lower phase (organic) layer was transferred to a new tube. The aqueous layer was extracted again with 1 mL of 2:1 chloroform/methanol. The chloroform layers were combined for analysis. Samples were then dried in a rotational vacuum concentrator, reconstituted in 50 μL of 1:1 isopropanol:acetonitrile and transferred into glass insert vials for LC-MS/MS analysis. To assess the performance of the analytical process and the suitable quality of the data, 10 μL aliquotes of all plasma samples were pooled as a quality control (QC) sample which was run at the beginning, middle and end of each batch.

**Untargeted lipid analysis by LC-MS/MS.** Lipid extracts were separated by an Ultra High Performance Liquid Chromatography (UHPLC) system (Ultimate 3000, Thermo) using a Reprosil C18 column (2.0 mm × 150 mm, 2.5 μm - Dr Maisch, Germany), mobile phases A (60% acetonitrile, 40% water, 10 mM ammonium acetate) and B (90% isopropanol, 10% water, 10 mM ammonium acetate). Samples were eluted at a flow rate of 250 μL/min with a linear gradient from 32% B to 97% B in 25 min, followed by 97% B for 4 min and 7 min re-equilibration with 32% B. The mass spectrometer (Q-Exactive from Thermo) operated in negative ionization mode with data dependent acquisition (DDA) mode for full-scan MS analysis. The full-scan settings were as follows: resolution, 70,000; automatic gain control (AGC) target, 3e6; maximum injection time (IT), 100 ms; and scan range, 250-3000. The remaining settings for DDA mode were as follows: resolution, 35,000; AGC target, 1e5; Isolation width 1.7 Da and collision energy (NCE), 30. Calibration was performed before each analysis against calibration mixes (Piercenet, Thermo Fisher, Rockford, IL) to ensure sub-ppm error of the intact mass. Lipid identification was performed with LipidSearch™ Search software.

**Metabolite extraction.** Plasma metabolites were extracted by adding 200 μL of each plasma sample to 600 μL of cold (−20 °C) chloroform:methanol:water (1:3:1 ratio). Samples were vortexed for 1 min and left on ice for 2 h for complete protein precipitation. The solutions were then centrifuged for 15 min at 15,000 × g for 10 min at 4 °C and the collected supernatants were dried. Finally, the dried samples were re-suspended in 0.1 mL of water 5% formic acid and transferred to glass autosampler vials for LC-MS analysis.

**Metabolite analysis by LC-MS.** Chromatographic separations were achieved by using a Reprosil C18 column (2.0 mm × 150 mm, 2.5 μm - Dr Maisch, Germany) at a temperature of 30 °C and a flow rate of 0.2 mL/min. A 0–100% linear gradient of solvent A (ddH2O, 0.1% formic acid) to B (acetonitrile, 0.1% formic acid) was employed over 20 min, returning to 100% A in 2 min and a 6-min post-time solvent A hold. The UHPLC system (Ultimate 3000, Thermo) was coupled online with a mass spectrometer Q-Exactive (Thermo) scanning in full MS mode (2 μscans) at 70,000 resolution in the 67 to 1000 m/z range, with a target of 1×10^6^ ions, maximum ion injection time (IT) of 35 ms. Source ionization parameters were: spray voltage, 3.8 kV; capillary temperature, 300 °C; sheath gas, 40; auxiliary gas, 25; S-Lens level, 45. Calibration was performed before each analysis against calibration mixes (Piercenet, Thermo Fisher, Rockford, IL) to ensure sub-ppm error of the intact mass. Raw files were exported, converted into mzXML format through MassMatrix (Cleveland, OH), and then processed by MAVEN software (http://maven.princeton.edu/). Results were graphed with Graphpad Prism 5.0 (Graphpad Software Inc).

**Table S1. Characteristics of study participants.** Data are presented as the mean ± SD. Statistical differences were determined using Tukey’s multiple comparisons. **p<0.01, ***p<0.001. IS, insulin-sensitive; TRT, testosterone replacement therapy

|  | **Control** | **IS hypogonadal men** | **IS hypogonadal men after TRT** | **p-value** | **Tukey HSD p-value** |
| --- | --- | --- | --- | --- | --- |
| **Subjects** | n-20 | n-20 | n-20 | - | - |
| **Age** | 42,54±13,67 | 42,18±16,01 | 42,18±16,01 | - | - |
| **BMI (Kg/m^2^)** | 24,91±4,01 | 25,44±3,04 | 25,24±3,09 | 0,93 | - |
| **Testosterone (nmol/l)** | 20,02±7,47 | 6,35±4,35 | 19,20±9,10 | 0,0001 ***p | (A vs B) 0,001 **p  (B vs C) 0,001 **p |
| **Glucose (mg/100ml)** | 84,72±4,38 | 81,45±12,51 | 86,90±6,77 | 0,3 | - |
| **Insuline (mUI/L)** | 7,77±3,13 | 6,72±2,88 | 6,99±2,98 | 0,69 | - |
| **HOMAi** | 1,79±0,86 | 1,97±0,67 | 1,47±0,70 | 0,92 | - |
| **Tg (mmol/l)** | 96,36±51,39 | 117,90±62,73 | 125,54±63,18 | 0,5 | - |
| **Cholesterol (mmol/l)** | 196,72±29,18 | 212,81±42,6 | 210,18±53,18 | 0,6 | - |
| **HDL Cholesterol (mmol/l)** | 53,90±11,98 | 52,63±15,01 | 47,45±15,47 | 0,54 | - |
| **LDL Cholesterol (mmol/l)** | 133,45±33,07 | 136,36±38,74 | 127,36±44,31 | 0,85 | - |

**Figure S1.** PCA scores plot of quality control (QC) samples generated by using MetaboAnalyst 4.0 software (<http://metpa.metabolomics.ca/>).


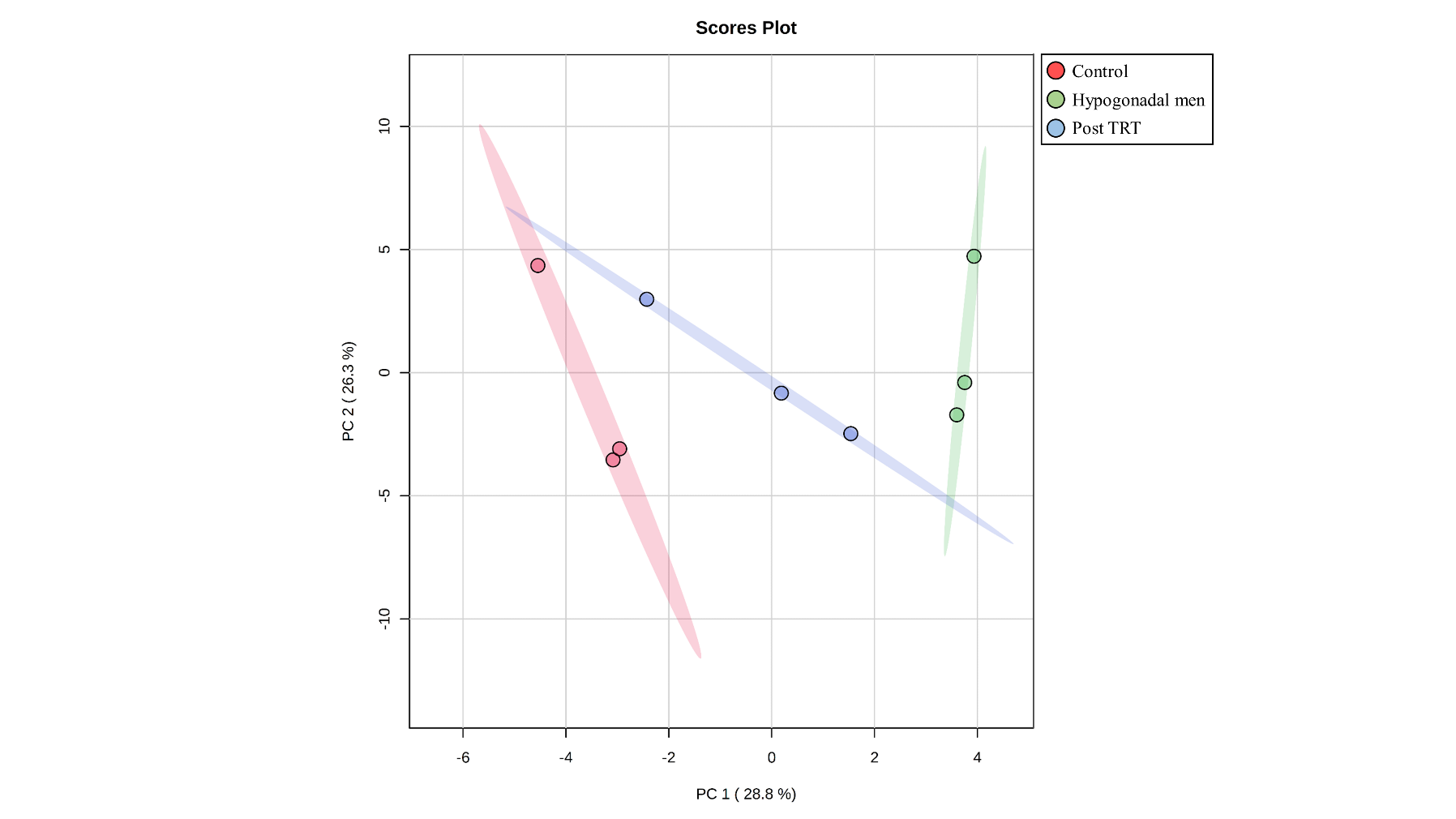

Supplement: Supplementary file 1 — Supplementary Material [file JCMM-24-8266-s001.docx]
